# Recombinant Decorin Fusion Protein Attenuates Murine Abdominal Aortic Aneurysm Formation and Rupture

**DOI:** 10.1038/s41598-017-16194-8

**Published:** 2017-11-20

**Authors:** Yue Shen, Valerio Russo, Matthew R. Zeglinski, Stephanie L. Sellers, Zhengguo Wu, Cameron Oram, Stephanie Santacruz, Yulia Merkulova, Christopher Turner, Keerit Tauh, Hongyan Zhao, Tatjana Bozin, Lubos Bohunek, Haishan Zeng, Michael A. Seidman, R. Chris Bleackley, Bruce M. McManus, Erkki Ruoslahti, Tero A. H. Järvinen, David J. Granville

**Affiliations:** 1 Centre for Heart Lung Innovation, St. Paul’s Hospital, University of British Columbia, Vancouver, BC Canada; 2 International Collaboration On Repair Discoveries (ICORD), Vancouver Coastal Health Research Institute and Department of Pathology and Laboratory Medicine, University of British Columbia, Vancouver, BC Canada; 3 Department of Radiology, University of British Columbia & St. Paul’s Hospital, Vancouver, BC Canada; 4 Imaging Unit, Integrative Oncology Department, BC Cancer Agency Research Centre, Vancouver, BC Canada; 5 Photomedicine Institute, Department of Dermatology and Skin Science, University of British Columbia & Vancouver Coastal Health Research Institute, Vancouver, BC Canada; 6 Department of Biochemistry, University of Alberta, Edmonton, AB Canada; 7 Cancer Research Center, Sanford-Burnham Prebys Medical Discovery Institute, La Jolla, CA 92037 USA; 8 Center for Nanomedicine and Department of Molecular Cellular and Developmental Biology, University of California, Santa Barbara, Santa Barbara, CA 93106-9610 USA; 9 Faculty of Medicine & Life Sciences, University of Tampere & Department of Orthopedics & Traumatology, Tampere University Hospital, Tampere, Finland; 10 PROOF Centre of Excellence, University of British Columbia & Providence Health Care, Vancouver, BC Canada

## Abstract

Decorin (DCN) is a small-leucine rich proteoglycan that mediates collagen fibrillogenesis, organization, and tensile strength. Adventitial DCN is reduced in abdominal aortic aneurysm (AAA) resulting in vessel wall instability thereby predisposing the vessel to rupture. Recombinant DCN fusion protein CAR-DCN was engineered with an extended C-terminus comprised of CAR homing peptide that recognizes inflamed blood vessels and penetrates deep into the vessel wall. In the present study, the role of systemically-administered CAR-DCN in AAA progression and rupture was assessed in a murine model. Apolipoprotein E knockout (ApoE-KO) mice were infused with angiotensin II (AngII) for 28 days to induce AAA formation. CAR-DCN or vehicle was administrated systemically until day 15. Mortality due to AAA rupture was significantly reduced in CAR-DCN-treated mice compared to controls. Although the prevalence of AAA was similar between vehicle and CAR-DCN groups, the severity of AAA in the CAR-DCN group was significantly reduced. Histological analysis revealed that CAR-DCN treatment significantly increased DCN and collagen levels within the aortic wall as compared to vehicle controls. Taken together, these results suggest that CAR-DCN treatment attenuates the formation and rupture of Ang II-induced AAA in mice by reinforcing the aortic wall.

## Introduction

Abdominal aortic aneurysm (AAA) is a common, age-related, life-threatening vascular pathology that affects approximately 9% of the population over 65 years of age^1^. AAA is usually asymptomatic until rupture, at which point, mortality rates as high as 90% have been reported^2^. There are no non-invasive therapeutics approved for preventing growth and/or rupture. Open surgical or endovascular repair remain as the only treatment options, and are only recommended at diameters above 5–5.5 cm at which point the risk of rupture surpasses the risk/complications associated with surgery. As such, any patients with small AAA experience anxiety due to this wait and watch approach. This has resulted in an increased impetus and need for effective non-invasive approaches to prevent or slow AAA progression^3^.

The small leucine rich proteoglycan, decorin (DCN) is ubiquitously expressed by numerous cell types, including fibroblasts and smooth muscle cells. DCN consists of a 40 kDa core protein that attaches to a single chondroitin/dermatan sulfate GAG chain. DCN elicits many roles in extracellular matrix homeostasis, including regulation of collagen fibrillogenesis^4^, collagen degradation^5^, cell signaling and cell growth^6–9^. Acting as an anchor between collagen fibrils, DCN provides elasticity and tensile strength to collagen fibers^10^. During collagen fibrillogenesis, DCN is also involved in fibril formation, fusion and organization^4^.

DCN is also a natural antagonist to transforming growth factor-β (TGF-β), a growth factor associated with AAA progression. Degradation of DCN during inflammation and remodeling increases the TGF-β bioavailability, which accelerates aortic aneurysm and may affect inflammation^11–14^. The murine serine protease inhibitor, Serpina3n (SA3N), prevents Granzyme B (GzmB)-mediated DCN degradation and improves collagen remodeling leading to a reduced aneurysm rupture rate and death in a murine model of AAA^15^.

By screening peptide libraries (1.0 × 10^9^) with *in vivo* phage display, we have identified a vascular homing peptide for the purpose of targeted delivery of systemically administered therapeutics to focal site with specificity^16^. The vascular homing peptide CAR (sequence CARSKNKDC) was originally identified as it recognizes angiogenic blood vessels and homes to the neo-vasculature in regenerating tissues^16–20^. Subsequent work established that CAR peptide also homes in to inflamed vessels in conditions associated with disrupted sheer stress/blood flow such as pulmonary arterial hypertension^17,18,20^. In addition to being a potent homing peptide, the CAR peptide is also a cell penetrating peptide capable of penetrating deep into surrounding parenchyma and thick arterial walls as well as delivering the pharmaceutical agents to target organ parenchyme^16–20^. We have generated a recombinant DCN fusion protein, CAR-DCN, where human DCN has been engineered with an extended C-terminus comprised of the CAR peptide^17^. The anchorage to cells afforded by CAR peptide in CAR-DCN has substantially increased its biological activity and systemically administrated CAR-DCN accumulated in the neo-vasculature-rich wound granulation tissue in significantly larger quantities than native DCN^17^. In addition to that, CAR-DCN is also substantially more active than the native DCN against TGF-β^17^. These features were associated with more rapid wound healing and suppressed scar formation when compared to improvement obtained by native DCN^17^.

AAA progression is associated with ongoing inflammation in concert with increased angiogenesis within the vessel wall^21^. Increased angiogenesis and expression of angiogenic, inflammatory cytokines are observed at the site of aneurysm rupture^22,23^. Based on our previous studies pertaining to the role of DCN in the development of AAA^15^, combined with the neo-vasculature- and inflammatory-homing characteristics of the CAR-DCN peptide prompted us to investigate whether systemic administration of CAR-DCN could affect the onset and progression of AAA. In the present study, CAR-DCN treatment was assessed using an angiotensin II (Ang II)-induced AAA model. We hypothesized that systemically-administered CAR-DCN would attenuate AAA progression and rupture, and increase survival.

## Results

### CAR-DCN Treatment Increases 28-day Survival and Reduces Severity of Ang II-induced AAA

**Figure 1:**
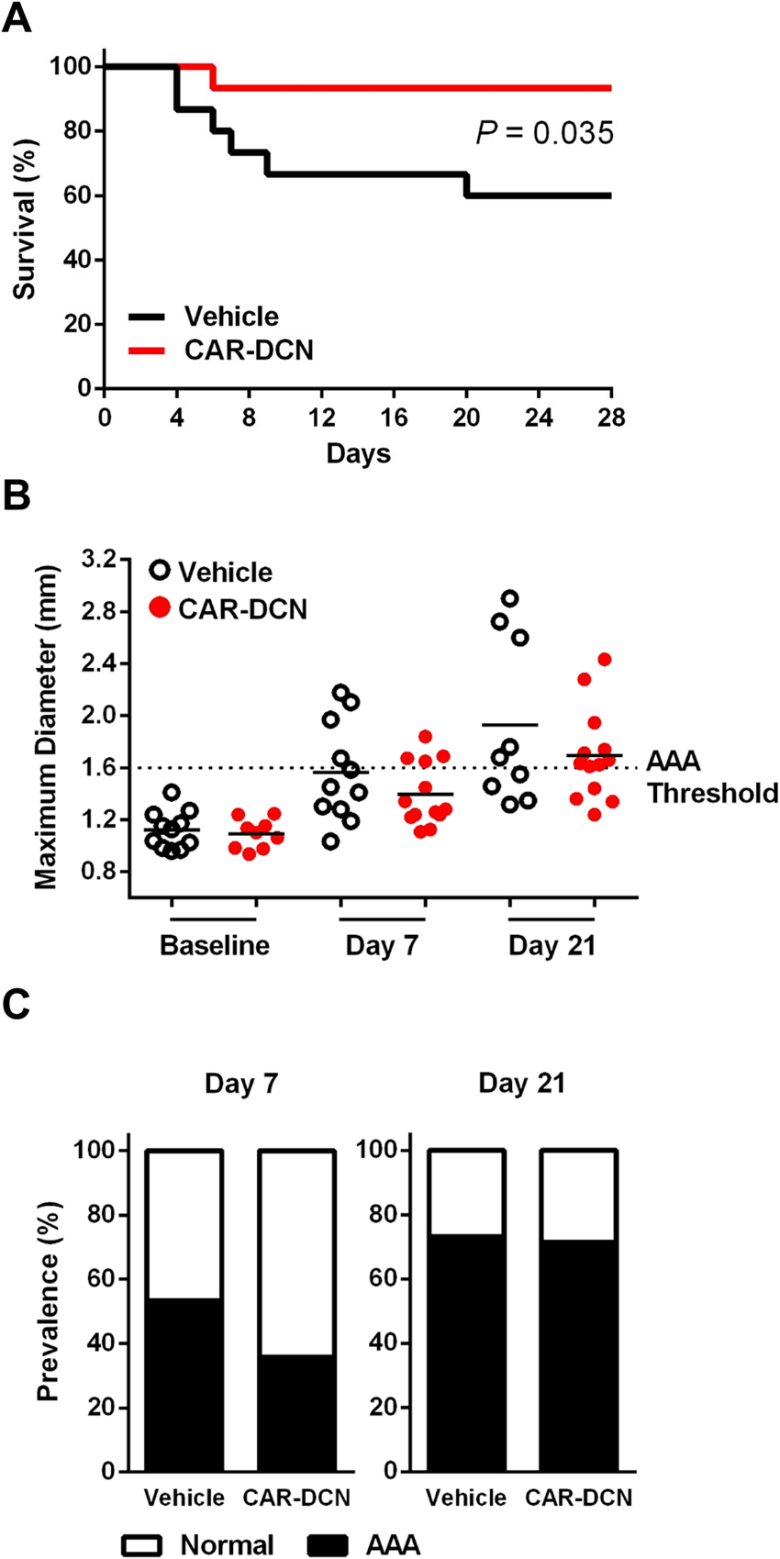
CAR-DCN treatment improves 28-day survival in Ang II-infused ApoE-KO mice. (**A**) 28-day survival rate in Ang II infused ApoE-KO mice treated with saline (60%, n = 15) or CAR-DCN (92.8%, n = 14). *P* = 0.035 by Log-rank (Mantel-Cox) test. **(B)** Scatter plot of maximum diameter of abdominal aorta in Ang II-infused ApoE-KO mice treated with vehicle or CAR-DCN. Solid line indicates the mean value of each group. Dash line indicates the AAA threshold (>50% enlargement of maximum aortic diameter in sham ApoE-KO mice). **(C)** AAA prevalence in Ang II-infused ApoE-KO mice treated with vehicle or CAR-DCN at day 7 and day 28, AAA is determined by ultrasonic measurements.

**Figure 2:**
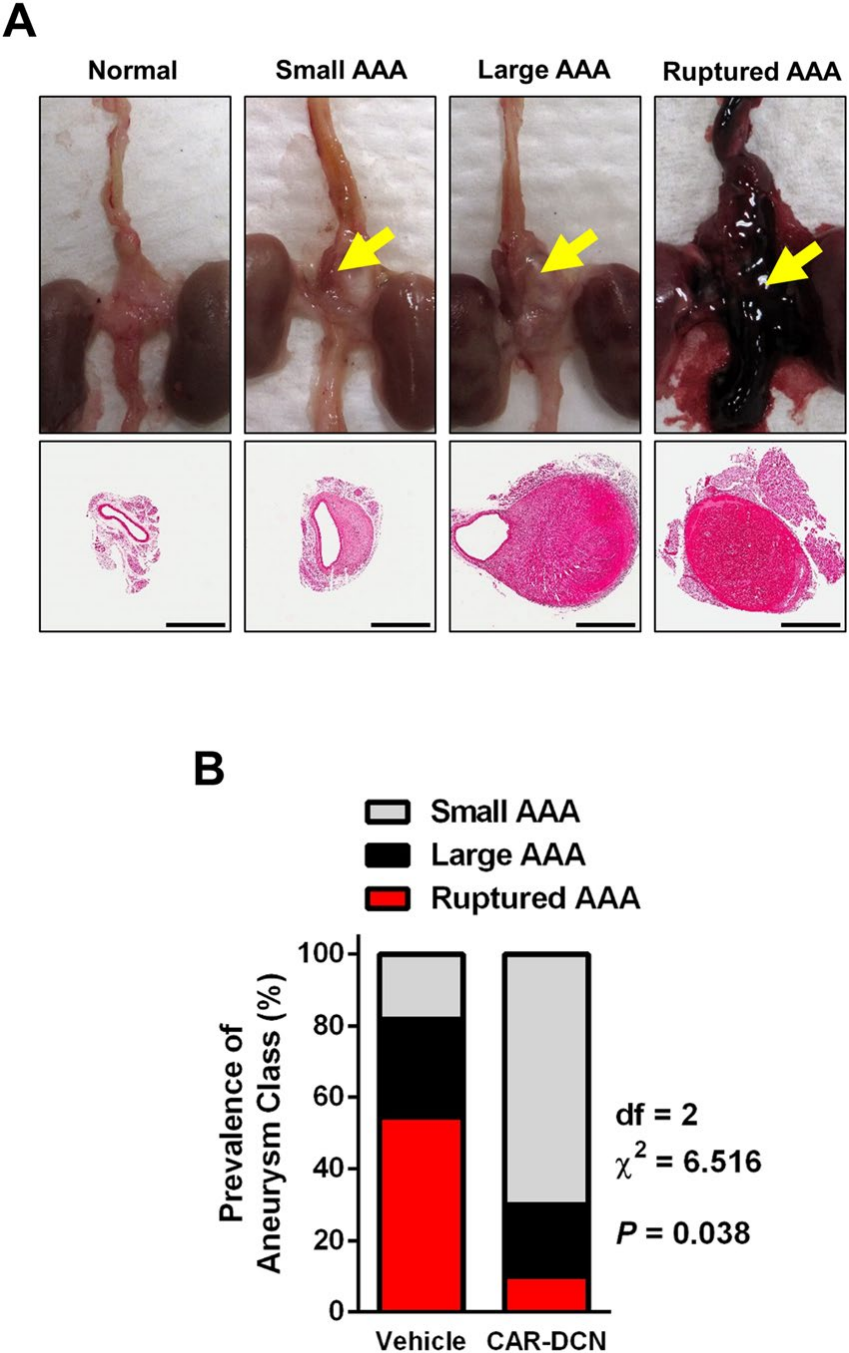
CAR-DCN treatment reduces AAA severity in Ang II-infused ApoE-KO mice. (**A**) Representative gross pathology and morphology of different aneurysm class. Yellow arrows denote aortic aneurysm. Aortas were stained with H&E to assess morphology. Scale bar: 1 mm. (**B**) Prevalence of aneurysm class in Ang II-infused ApoE-KO mice treated with vehicle (n = 15) or CAR-DCN (n = 14), *P* = 0.038 by chi-square test.

CAR-DCN-treated, Ang II-infused ApoE-KO mice exhibited a significant increase in 28-day survival (92.8%, n = 14; vs 60%, n = 15; *P* = 0.035) in comparison to vehicle-treated controls (Fig. 1A). Necropsy was performed on all mice that died before the 28-day time point to determine cause of death. All cases of premature death exhibited signs of exsanguination in the abdominal cavity indicative of AAA rupture. To follow AAA progression, all mice were examined by ultrasound at three time points (day 0 prior to pump implantation, and day 7 and day 21 post-implantation). AAA was defined as a >50% enlargement of the maximum aortic diameter in sham ApoE-KO mice, in line with the current clinical definition^24^. The CAR-DCN treated group exhibited a smaller average maximum aortic diameter at both day 7 (1.39 ± 0.07, n = 13; vs 1.56 ± 0.11, n = 11) and day 21 (1.69 ± 0.10, n = 13; vs 1.93 ± 0.21, n = 9) when compared with the vehicle control group (Fig. 1B), however the difference did not reach statistical significance. The onset of AAA was similar between CAR-D CN and vehicle groups (Fig. 1C). All aortas were harvested and histologically assessed for aneurysm. Based on previously published criteria^25^, aneurysms were categorized into three classes: small AAA, large AAA and ruptured AAA (Fig. 2A). Among all mice that developed AAA, greater than 50% of vehicle-treated mice exhibited an aneurysmal rupture (54.5%, n = 6/11) compared to only 10% of CAR-DCN-treated mice (10%, n = 1/10) (Fig. 2B). Although the prevalence of large but non-ruptured aneurysm was similar between vehicle and CAR-DCN groups (27.2%, n = 3/11; vs 20%, n = 2/10), the majority of aneurysms in CAR-DCN group were small AAAs (70%, n = 7/10 vs. 18% in vehicle control, n = 2/11) (Fig. 2B). The prevalence of aneurysm was analyzed using a chi-square test and a statistically significant difference was observed between the vehicle control group and CAR-DCN group (*P* = 0.038).

### CAR-DCN Treatment Increases Adventitial Collagen Organization

**Figure 3:**
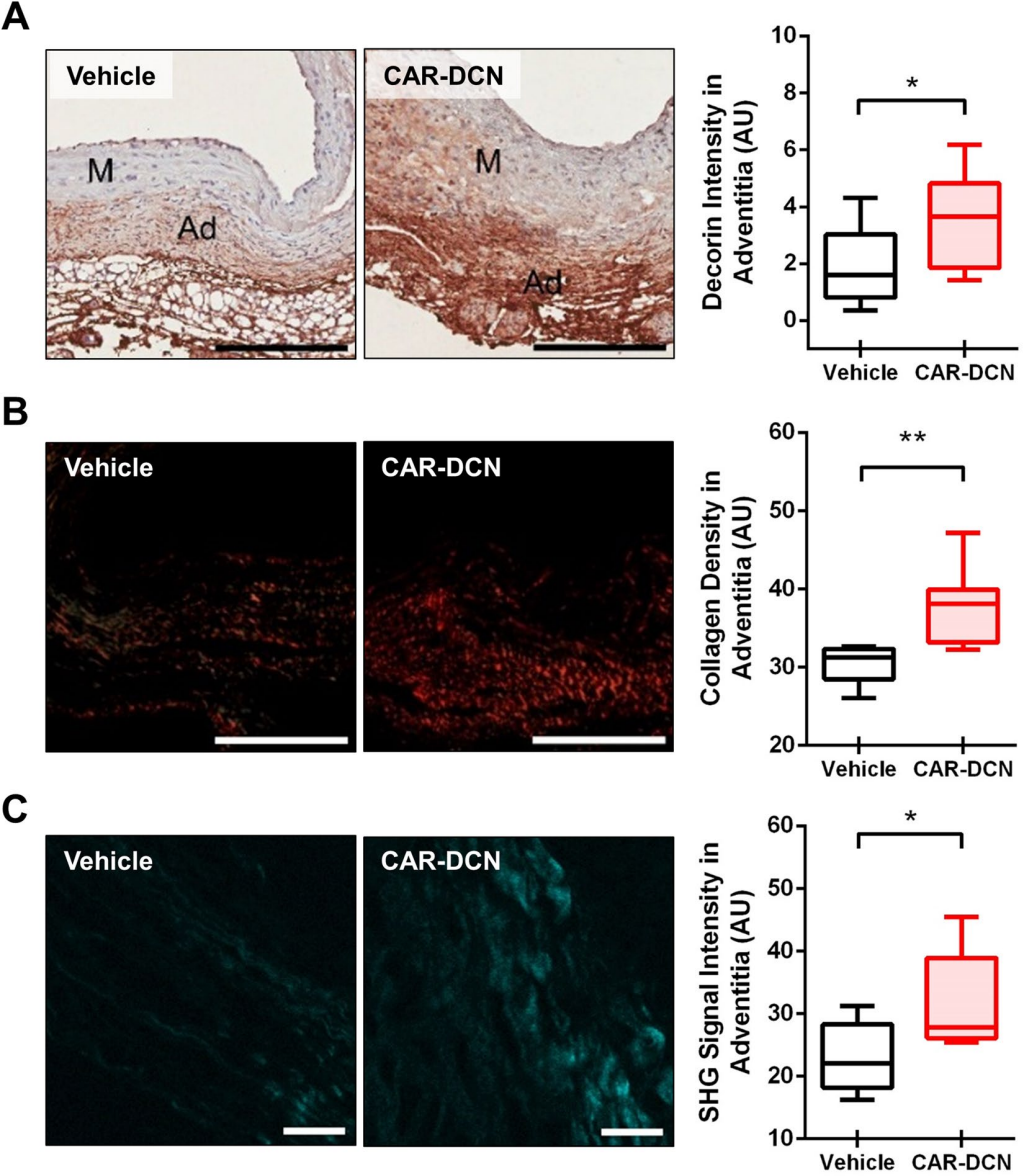
CAR-DCN treatment increases adventitial DCN and adventitial collagen organization. Representative (**A**) decorin and (**B**) picrosirius red staining (left) and quantification of (**A**) decorin and (**B**) picrosirius red staining intensity (right) in abdominal aortas from of surviving ApoE-KO mice in different treatment groups (n ≥ 6 per group). M, media; Ad, adventitia; Scale bar: 200 μm. (**C**) Representative SHG imaging (left) and quantification of SHG signal intensity (right) in adventitia of abdominal aortas from surviving ApoE-KO mice in different treatment groups (n ≥ 6 per group). Scale bar: 20 μm. Results are expressed as box-and-whisker plot, *P < 0.05, **P < 0.01 by Student t-test.

Adventitial DCN was markedly higher in the CAR-DCN group as compared to vehicle controls (Fig. 3A). Adventitial collagen was assessed using picrosirius red staining, collagen fibers from the vehicle control group stained green and yellow, indicating a weaker and immature collagen structure that was consistent with previous studies^15^. Conversely, collagen fibers from the CAR-DCN group appeared to be mostly orange and red, suggesting a more mature, organized collagen structure (Fig. 3B). These findings were confirmed using second harmonic generation (SHG) collagen analysis (Fig. 3C). To confirm whether CAR peptide homes to the adventitia in AAA, a peptide homing study was performed using fluorescent-labelled peptides. Both CAR and mutant CAR peptide were i.v.-administered into these animals to determine peptide homing to AAA. The strong green autofluorescence of the abdominal aorta and the penetration of CAR peptide to parenchyme make it difficult to accurately interpret the data of direct FAM imaging. For this reason, and to preserve the histologic features, we used IHC staining with anti-fluorescein antibodies to detect the FAM-labeled peptides. Strong accumulation of CAR was observed in the aortic adventitia both in blood vessels and the surrounding parenchyme (Supplemental Figure S1).

### CAR-DCN Treatment Does Not Affect Medial Disruption

**Figure 4:**
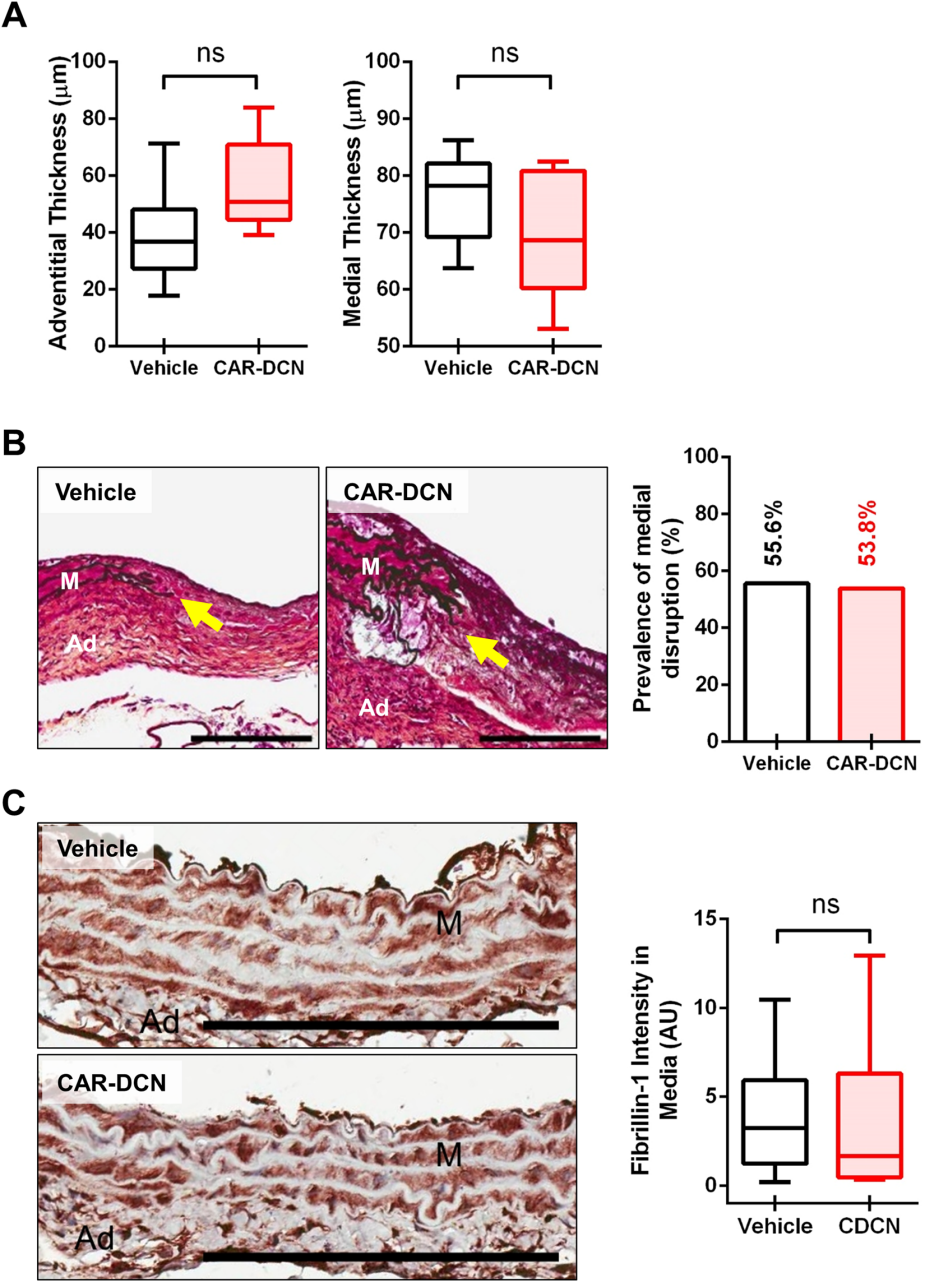
CAR-DCN treatment does not protect against medial disruption. (**A**) Quantification of adventitial and medial thickness in abdominal aortas of ApoE-KO mice in different treatment groups (n ≥ 6 per group). (**B**) Representative Movat’s pentachrome staining (left) and prevalence of medial disruption (right) in abdominal aortas from surviving ApoE-KO mice in different treatment groups. Arrows indicate medial disruption. M, media; Ad, adventitia; Scale bar: 200 μm. (**C**) Representative fibrillin-1 staining (left) and quantification of fibrillin-1 staining intensity (right) in tunica media from of surviving ApoE-KO mice in different treatment groups (n ≥ 6 per group). M, media; Ad, adventitia; Scale bar: 200 μm. Results are expressed as box-and-whisker plot, ns = not significant.

Although the CAR-DCN group also showed an increase in the average adventitial thickness as compared to the vehicle control group (55.8 µm in CAR-DCN group vs 39.0 µm in vehicle control group), the difference was not statistically significant (Fig. 4A). The medial thickness of CAR-DCN and vehicle groups was comparable (Fig. 4A). Severe elastic fiber fragmentation was observed in both CAR-DCN and vehicle groups and the prevalence of medial disruption between these two groups were similar, indicating that CAR-DCN treatment did not prevent medial disruption (Fig. 4B). Fibrillin-1 levels in the tunica media were also similar between vehicle and CAR-DCN groups (Fig. 4C). GzmB was detected in the aortas of mice that exhibited AAA, and there was no difference on the level of GzmB staining between vehicle control and CAR-DCN groups (Supplemental Figure S2A). To examine whether CAR-DCN is subject to GzmB-mediated cleavage, a GzmB-CAR-DCN cleavage assay was performed. GzmB reduced full-length CAR-DCN levels, which was abolished by inhibition of GzmB with SA3N (Supplemental Figure S2B).

## Discussion

Aortic aneurysm is a leading cause of death in North America^26^. Smoking, male gender, aging and family history are the major risk factors for AAA^26,27^. The risk of AAA increases dramatically after 60 years of age^28^. Currently, the only treatment options available are elective open surgical repair, which is associated with a 5.6% operative mortality, or endovascular repair, which is associated with increased risk of post-surgery rupture. To date, no approved, non-surgical interventions are available^28^. The present study suggests that systemic administration of CAR-DCN, a target tissue-specific non-surgical intervention, can reduce AAA severity and rupture by increasing adventitial collagen organization.

Previous studies have demonstrated that inhibition of GzmB-mediated adventitial DCN degradation leads to increased collagen density and reduced aneurysm rupture^15^. In the present study, we tested an alternative approach by compensating the loss of DCN in AAA through exogenous DCN supplementation. A 15-day regimen was determined based on previous studies in which the vast majority of ruptures in Ang II-induced AAA occurred within the first two weeks of Ang II infusion^15,25,29,30^. CAR peptide is a cell/tissue penetrating peptide that has also been shown to specifically accumulate in the adventitia in a murine model of pulmonary arterial hypertension^18,19^. In the present study, adventitial DCN content was significantly increased. Additionally, collagen thick bundle formation was greater with CAR-DCN treatment as comparison to vehicle controls. Although CAR-DCN treatment did not affect aneurysm onset, aneurysm severity was significantly attenuated based on 28-day survival, ultrasound measurements and pathology examination.

One possible limitation of CAR-DCN treatment compared to previous AAA studies where GzmB was deficient or inhibited^15,25^ is that CAR-DCN treatment did not prevent medial disruption or change GzmB levels. Our *in vitro* data further showed that CAR-DCN is susceptible to GzmB-mediated cleavage. During aneurysm progression, GzmB cleaves many extracellular matrix proteins including DCN and fibrillin-1 both of which play pivotal roles in maintaining vessel wall stability^15,25^. Despite a significant attenuation of AAA progression, CAR-DCN treatment did not appear to affect other matrix proteins such as fibrillin-1 that are subject to GzmB-mediated proteolysis. This raises a possibility of further re-engineering CAR-DCN whereby the GzmB cleavage site is mutated to be resistant to cleavage.

The majority of human AAA samples are collected from autopsy or elective surgery, at which point the stage of aneurysm is considered to be advanced, late stage lesions. Currently, there are several animal models of aortic aneurysm available of which the Ang II AAA model is the most widely used. These animal models of AAA replicate many of the cellular and biochemical characteristics of the human disease, including a chronic inflammatory response and extensive extracellular matrix degradation and are severe enough to be eventually lethal^29,31,32^. Although animal models of AAA display certain features that are distinct from what is seen in human AAA samples, they allow us to investigate the initial pathological changes of the disease and examine the early interventions. Similar to what we have seen in the past^15^ and in the present study, a recent study from Ueda *et al*. confirmed that the level of DCN decreased at the early stage of disease. In addition, they further showed that implantation of exogenous DCN containing Gel foam patches in the periaortic space prevented the development of AAA^33^. In the present study, we used a novel protein CAR-DCN, which is more active than native DCN against TGF-β, an important player in abdominal aneurysm^17^. Furthermore, as opposed to previous studies^33^, in which surgical implantation of gelfoam patches containing DCN was utilized to localize DCN to the periaortic space, a non-invasive, more clinically-viable approach, i.e. systemic administration of CAR-DCN, was applied. As such, given similar results were observed, our studies support the systemic administration of CAR-DCN as a clinically viable, non-surgical intervention for early/small AAA treatment.

Taken together, the current study demonstrates the utility of a novel therapeutic approach to attenuate AAA progression through systemic administration of CAR-DCN. In conclusion, our findings support the future development of an AAA therapy involving systemic administration of CAR-DCN to delay and/or prevent aneurysmal rupture.

## Materials and Methods

### Mice

Apolipoprotein E knockout (ApoE-KO) male mice (C57Bl/6 background, Stock No. 002052) were obtained from Jackson Laboratories, Bar Harbor, ME, USA. All mice were housed at The Genetic Engineered Models facility at St. Paul’s Hospital, University of British Columbia. All procedures were performed in accordance with the guidelines for animal experimentation approved by the Animal Experimentation Committee of the University of British Columbia.

### Angiotensin II–Induced AAA

AAA was induced by Ang II infusion, as previously described^15^.

### Recombinant Protein Production

Recombinant CAR-DCN was expressed in 293-F cells using the FreeStyle 293 expression system as described previously^16^. Briefly, a pcDNA3.1/myc-his-C plasmid encoding for CAR-DCN was mixed with Opti-MEM I media and 293fectin transfection reagent. The DNA:293fectin solution was added to 293-F cells and the cells were cultured for 48 h. Recombinant protein was isolated from the media utilizing poly-histidine tag binding on Ni-NTA agarose beads (Qiagen) using 5 mL of beads per 500 mL of media. After an overnight incubation at 4 °C, the beads were washed with PBS, and CAR-DCN was eluted with PBS containing 300 mM imidazole, dialyzed against PBS, and stored at −80 °C.

### CAR-DCN Treatment

Ang II-infused ApoE-KO mice were given a tail vein injection of vehicle control (PBS) or CAR-DCN (40 μg/100 μL PBS per injection) every third day until day 15. Intraperitoneal injection of CAR-DCN or vehicle control was performed once a day between tail vein injections. The CAR-DCN treatment regimen is shown in Supplemental Figure S2. The dose for the treatment was selected on the basis of previous studies^17^.

### *In Vivo* Ultrasound Measurement

Abdominal aortas of the mice were visualized using a VisualSonics Vevo 2100 High-Resolution Imaging System with a 40-MHz frequency transducer (Fujifilm VisualSonics, Toronto, ON, Canada) before the implantation and at day 7 and day 21 post-implantation, as previously described^34^.

### Tissue Collection and Preparation

Four weeks following implantation, tissues from surviving mice were harvested as previously described^15^. Aortic segments were isolated from the abdominal aorta immediately above the renal arteries and stored in 10% formalin overnight before embedding in paraffin and sectioning. Necropsies were performed on all mice that died before the 4-week time point to determine the cause of death.

### Histological Analysis

Abdominal aortic sections were stained with Movat’s pentachrome and picrosirius red. Immunohistochemistry was performed using rabbit anti-mouse Granzyme B (Abcam, Cambridge, MA, USA), rabbit anti-Fibrillin-1 (Abcam), rabbit anti-fluorescent (ThermoFisher, St. Louis, MO, uSA) and goat anti-mouse decorin (R&D Systems, Minneapolis, MN, USA) as described previously^15^. Histological evaluation for severity of disease was performed independently by two experimental pathologists who were blinded to the experimental conditions.

### Second Harmonic Generation Microscopy and Collagen Analysis

Collagen in the vessel wall was visualized using a customized video rate multimodality multiphoton microscopy system^35^. Image acquisition was carried out at 15 frames per second with a resolution of 512 by 512 pixels. The laser source was an 80 MHz Ti:Sappire femtosecond laser (Chameleon, Coherent Inc., Santa Clara, CA, USA) with a wavelength tuning range of 700 nm-950 nm. The fast imaging speed was realized by using an 8 kHz resonance scanner for the fast axis and a galvanometer scanner for the slow axis. A 60X (NA = 1.0) water-immersion objective (LUMPLFLN60X/W, Olympus Canada, Markham, ON, Canada) was used to focus the laser light into the sample. The second harmonic signal was collected in the epi-direction by the same objective and was then reflected by a dichroic mirror (FF665-Di02–25 × 36, Semrock, Inc., Rochester, NY, USA) and focused into a photomultiplier tubes (PMT, H9433MOD-03, Hamamatsu Corp., Bridgewater, NJ, USA). A band pass filter (FF01–390/40–25, Semrock, Inc.) was located in front of the PMT with a transmission range from 370 nm to 410 nm for SHG detection with an excitation wavelength of 800 nm. Acquired images were averaged every 10 frames to improve the signal to noise ratio. The total SHG signal intensity values were quantified by ImageJ 1.5i.

### CAR-DCN Cleavage Assay

CAR-DCN (5 µg) was incubated with 200 nM of human GzmB (Beryllium, Boston, MA, USA) overnight at 37 °C in a water bath in digestion buffer (100 mM HEPES pH 7.5, 0.20% w/v CHAPS, 10 mM DTT). To demonstrate the functionality of GzmB to cleave CAR-DCN, we pre-incubated 200 nM of GzmB (Beryllium) with 600 nM of Serpin A3N (SA3N), (a generous gift from Dr. Chris R. Bleackley, University of Alberta, Edmonton, AB, Canada) for 60 minutes at 37 °C in a water bath. After pre-incubation, 5.0 µg of CAR-DCN was added to each reaction and incubated overnight at 37 °C in a water bath. After incubation, proteins were denatured and separated on a 10% SDS- polyacrylamide gel. The gel was stained by SimplyBlue Safe Stain (Invitrogen, Burlington, ON, Canada) and imaged using the LICOR Odyssey Fc (LI-COR Biotechnology, Lincoln, NE, USA) under the 600 channel with a 2 minute acquisition cycle. Image was then pseudocoloured to black on white for easier visualization. Fibronectin (92784, Abcam) was also used in this assay as positive control.

### Peptide Targeting Study

The following peptides labeled with FITC or 5-carboxyfluorescein (FAM) were used for the aorta targeting studies: CAR, CARSKNKDC and CAR mutant (control peptide), CAQSNNKDC. Peptides were dissolved in PBS at concentrations of 0.5 mg/mL. ApoE-KO mice were injected with peptide solution through the tail vein (3 mg/kg) three days after AngII infusion. Two hours after injection, the mice were perfused with PBS containing 1% bovine serum albumin while under deep anesthesia, and tissues were fixed by systemic perfusion with 10% buffered formalin. The aortas were excised and fixed for an additional 24 hours and processed for anti-fluorescein immunohistochemistry (IHC) analysis as described previously^18,19^.

### Statistical Analysis

Quantitative values are expressed as mean ± SEM. Statistical analysis was performed using GraphPad Prism version 5.01 (GraphPad Software, San Diego, CA, USA). Survival curves were assessed using Log-rank test for trend and Log-rank/Mantel-Cox analysis. Prevalence of AAA class was assessed by chi-square test. Decorin intensity, collagen density and SHG signal intensity was assessed by unpaired Student’s *t*-test. For all tests, significant difference were set at *P* < 0.05.

### Data Availability

All data generated or analysed during this study are included in this published article (and its Supplementary Information files).

## Electronic supplementary material

Supplemental Information
