# Supplementary material for: Recombinant Decorin Fusion Protein Attenuates Murine Abdominal Aortic Aneurysm Formation and Rupture
Source: Sci Rep. 2017 Nov 20;7:15857. doi: 10.1038/s41598-017-16194-8 (PMC5696466; doi:10.1038/s41598-017-16194-8)
Supplement: Supplementary file 1 — Supplemental Information [file 41598_2017_16194_MOESM1_ESM.pdf]

SUPPLEMENTAL DATA

Recombinant Decorin Fusion Protein Attenuates Murine Abdominal Aortic Aneurysm Formation and Rupture

Authors and affiliations:

Yue Shen[1,2], Valerio Russo[1,2], Matthew R. Zeglinski[2], Stephanie L. Sellers[1,3], Zhengguo Wu[4,5], Cameron Oram[1,2], Stephanie Santacruz[1,2], Yulia Merkulova[1,2], Christopher Turner[1,2], Keerit Tauh[2], Hongyan Zhao[1,2], Tatjana Bozin[1], Lubos Bohunek[1], Haishan Zeng[4,5], Michael A. Seidman[1], R. Chris Bleackley[6], Bruce M. McManus[1,10], Erkki Ruoslahti[7,8], Tero A. H. Järvinen[9], and David J. Granville[1,2]*

[1]Centre for Heart Lung Innovation, St. Paul's Hospital, University of British Columbia, Vancouver, BC, Canada; [2]International Collaboration On Repair Discoveries (ICORD), Vancouver Coastal Health Research Institute and Department of Pathology and Laboratory Medicine, University of British Columbia, Vancouver, BC, Canada; [3]Department of Radiology, University of British Columbia & St. Paul's Hospital, Vancouver, BC, Canada; [4]Imaging Unit, Integrative Oncology Department, BC Cancer Agency Research Centre, Vancouver, BC, Canada; [5]Photomedicine Institute, Department of Dermatology and Skin Science, University of British Columbia & Vancouver Coastal Health Research Institute, Vancouver, BC, Canada; [6]Department of Biochemistry, University of Alberta, Edmonton, AB, Canada; [7]Cancer Research Center, Sanford-Burnham Prebys Medical Discovery Institute, La Jolla, CA 92037, USA; [8]Center for Nanomedicine and Department of Molecular Cellular and Developmental Biology, University of California, Santa Barbara, Santa Barbara, CA 93106-9610, USA; [9]Faculty of Medicine & Life Sciences, University of Tampere & Department of Orthopedics & Traumatology, Tampere University Hospital, Tampere, Finland. [10]PROOF Centre of Excellence, University of British Columbia & Providence Health Care, Vancouver, BC, Canada.

***Correspondence:**

David J Granville, ICORD Centre, Rm 4470, 818 West 10th Ave.
Vancouver, BC, V5Z 1M9, CANADA. Tel: +1-604-806-9267; Fax: +1-604-806-8351; Email. dgranville@icord.org

Control peptide

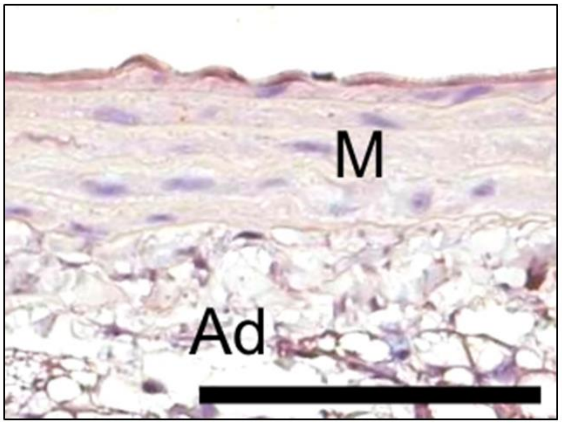

CAR peptide

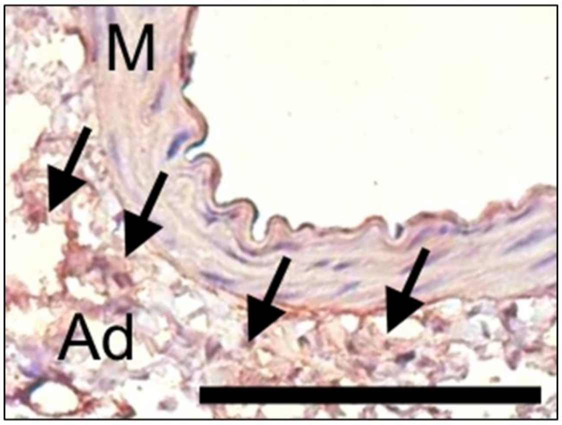

Supplemental Figure S1

Supplemental Figure S1. CAR peptide accumulates at adventitia layer of abdominal aorta. Representative FITC/FAM staining of adventitia in abdominal aortas from Ang II-infused ApoE-KO mice treated with FAM-labelled control peptide (upper) or FITC-labelled CAR peptide (lower). Arrows indicate the positive CAR staining. Scale bar: 100 μm.

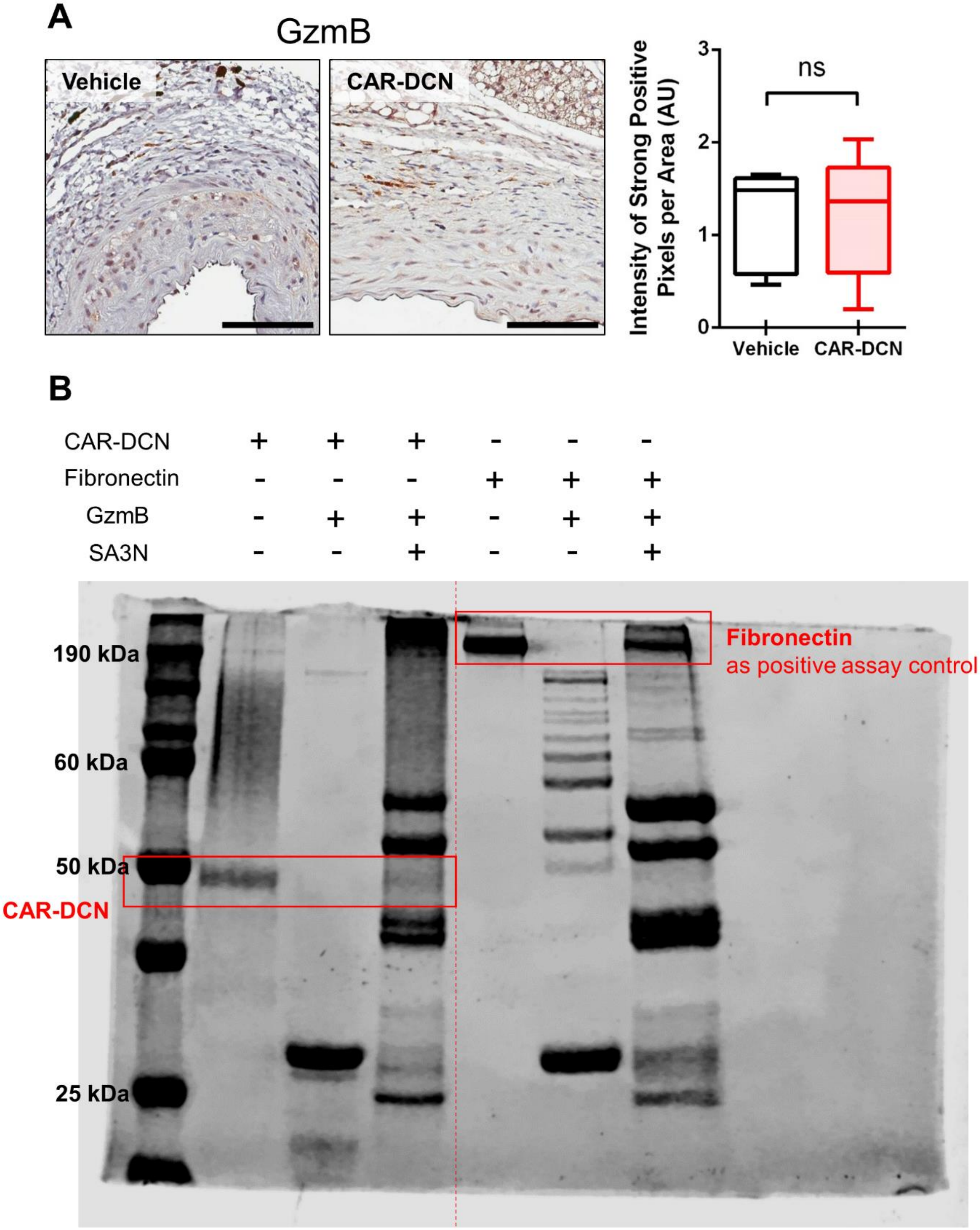

Supplemental Figure S2

Supplemental Figure S2. CAR-DCN treatment does not affect aortic GzmB expression. (A) Representative GzmB staining (left) and quantification of GzmB staining intensity (right) in adventitia of abdominal aortas from surviving ApoE-KO mice in different treatment groups ($n \geq 5$ per group). Scale bar: 100 μm. Results are expressed as box-and-whisker plot, ns = not significant. **(B)** GzmB-mediated CAR-DCN cleavage assay. The presence of SA3N inhibits GzmB-mediated CAR-DCN cleavage. Fibronectin was used as positive control.

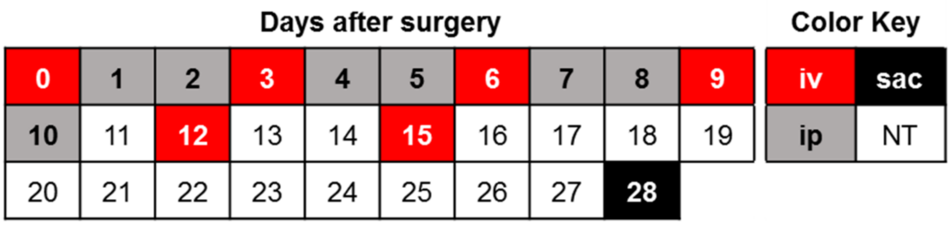

Supplemental Figure S3

Supplemental Figure S3. Treatment Regimen. Paradigm for regimen of CAR-DCN or vehicle administration in Ang II-infused ApoE-KO mice. iv, intravenous injection; ip, intraperitoneal injection; sac, sacrifice; NT, no treatment.
